# The Dynamics of Information Search Queries about COVID-19 at the Self-Isolation Stage

**DOI:** 10.1192/j.eurpsy.2022.1374

**Published:** 2022-09-01

**Authors:** E. Belinskaya, O. Tihomandritskaya, E. Stolbova, E. Tsikina

**Affiliations:** Lomonosov Moscow State University, Faculty Of Psychology, Moscow, Russian Federation

**Keywords:** global threat, search information query, affective states

## Abstract

**Introduction:**

The dynamics of search queries in large random samples may reflect both the reaction of the population to the statements of power subjects, and the presence of certain mechanisms of emotional self-regulation.

**Objectives:**

Studying the dynamics of information search queries in the situation of experiencing an objective global threat-the spread of COVID-19.

**Methods:**

Quantitative methods of the Data Mining class were used for data collection. Qualitative methods of the Data Mining class and contextual analysis were used for data analysis. Search queries related to COVID-19 and the introduction of self-isolation were analyzed: 5 million queries were randomly selected.

**Results:**

The number of information requests on the topic of the pandemic increased sharply after V. Putin’s first address, and then steadily decreased, but the overall picture of the content dynamics of search queries, depending on official statements of the authorities, was not observed; requests about the current level of morbidity are constantly presented to the greatest extent, and to the minimum-about the possibilities of psychological assistance and the state of the Russian economy. During the whole time of the study the contents of the Internet search shifted towards positive emotional information.

**Conclusions:**

. To the maximum extent, the relationship between the experience of a pandemic and self-isolation with the nature of search queries manifests itself at the initial stages and tends to decrease in the future. The dynamics of the content of requests is ambiguously related to official statements of power subjects, influenced by the effect of counter-regulation.

**Disclosure:**

No significant relationships.

